# Study of Different Deep Learning Methods for Coronavirus (COVID-19) Pandemic: Taxonomy, Survey and Insights

**DOI:** 10.3390/s22051890

**Published:** 2022-02-28

**Authors:** Lamia Awassa, Imen Jdey, Habib Dhahri, Ghazala Hcini, Awais Mahmood, Esam Othman, Muhammad Haneef

**Affiliations:** 1 Faculty of Sciences and Technology of Sidi Bouzid, University of Kairouan, Kairouan 3100, Tunisia; lamiaawassa2020@gmail.com (L.A.); imen.jdey@fstsbz.u-kairouan.tn (I.J.); hcinighazala@fstsbz.u-kairouan.tn (G.H.); 2 Department of Information Science, College of Applied Computer Sciences, King Saud University, Riyadh 11451, Saudi Arabia; mawais@ksu.edu.sa (A.M.); eothman@ksu.edu.sa (E.O.); 3 Department of Electrical Engineering, Foundation University Islamabad, Islamabad 44000, Pakistan; muhammadhaneef@fui.edu.pk

**Keywords:** COVID-19, deep learning, diagnostic, computer tomography (CT), chest X-rays (CXR), classification

## Abstract

COVID-19 has evolved into one of the most severe and acute illnesses. The number of deaths continues to climb despite the development of vaccines and new strains of the virus have appeared. The early and precise recognition of COVID-19 are key in viably treating patients and containing the pandemic on the whole. Deep learning technology has been shown to be a significant tool in diagnosing COVID-19 and in assisting radiologists to detect anomalies and numerous diseases during this epidemic. This research seeks to provide an overview of novel deep learning-based applications for medical imaging modalities, computer tomography (CT) and chest X-rays (CXR), for the detection and classification COVID-19. First, we give an overview of the taxonomy of medical imaging and present a summary of types of deep learning (DL) methods. Then, utilizing deep learning techniques, we present an overview of systems created for COVID-19 detection and classification. We also give a rundown of the most well-known databases used to train these networks. Finally, we explore the challenges of using deep learning algorithms to detect COVID-19, as well as future research prospects in this field.

## 1. Introduction

The Wuhan Municipal Health Commission initially reported a substantial concentration of pneumonia patients in Wuhan City, Hubei Province, China (World Health Organization (WHO), 2020) on 31 December 2019.The virus, known as SARS-CoV-2 (severe acute respiratory syndrome coronavirus 2), can cause severe pneumonia and has been shown to spread from person to person [1].

In order to deal with the spread of COVID-19, effective screening and early medical care for affected people are critical requirements. The most commonly utilized clinical screening approach for COVID-19 patients is reverse transcription polymerase chain reaction (RT-PCR), which employs respiratory materials for testing. However, RT-PCR has a poor diagnostic sensitivity, often necessitates multiple tests to confirm infection, and is very time-consuming [2]. To address this problem, an alternative diagnostic approach, based on screening chest radiography images (CRIs), such as X-ray or computed tomography (CT) images, is being developed, as COVID-19 patients frequently exhibit aberrant lung infection characteristics on CRIs [3].

Clinical symptom analyses, epidemiological history, positive radiographic imaging (computed tomography (CT)/chest radiograph (CXR)), and positive pathogenic tests are among the COVID-19′s other diagnostic procedures [4].

### 1.1. COVID-19

COVID-19 was declared a global pandemic by the World Health Organization (WHO) in March, 2020 [5]. COVID-19, often known as SARS-CoV-2, is a novel virus in the severe acute respiratory syndrome coronavirus (SARS-CoV) family. Over time, viruses continue to change due to mutations and new virus variants will appear. Sometimes new variants appear and then disappear. Other times, new variations appear and continue to exist. In recent months, several new strains of SARS-CoV-2 have emerged. In the fall of 2020, the United Kingdom (UK) discovered a variant named B.1.1.7 with a substantial number of mutations [6]. Apart from B.1.1.7, another version known as 501Y.V2 or B.1.351 arose in South Africa [7]. B.1.351 and B.1.1.7 have certain mutations in common. In early January, a new strain known as P.1 was discovered in Brazilian travelers who were tested during standard screening at a Japanese airport [8].

In India, a new COVID variant called B.1.617 was first detected in December 2020. B.1.617 is a variation that has had a major impact on the second influx of contaminations in India, and has spread to numerous different nations, including the UK. Between the end of 2020 and the time of writing this paper, several other strains have been identified [9]. SARS-CoV-2 mutations are causing concern all over the world. Indeed, some variants are more contagious and have a higher transmission rate than prior ones [10,11], resulting in an increase of COVID-19 patients. In general, almost all coronavirus variants can be distinguished only by sequencing the genome of the virus. Moreover, despite the discovery of vaccinations against SARS-CoV-2/COVID-19 worldwide, this virus is likely to continue to evolve, which makes controlling it more difficult [12]. The rising number of cases will put more strain on medical services, potentially leading to more hospitalizations and fatalities. It is thus critical to recognize those who are infected. The introduction of automatic detection systems based on AI has shown an encouraging effectiveness of artificial intelligence (AI) in detecting numerous types of malignancies during the large expansion of the COVID-19 outbreak, as these can lead to a quick diagnosis of infected cases and assist in their rapid isolation [13]. Several AI-based solutions have been developed to make COVID-19 detection and decision making in medical image screening faster and more accurate. The use of a deep learning algorithm to perform image classification is an essential element of study disciplines. The implementation of deep learning in the COVID-19 pandemic has resulted in improved disease diagnosis and classification based on both X-ray and CT imaging.

### 1.2. Taxonomy of Medical Imaging

Clinical studies have shown that the majority of COVID-19 patients, during the current coronavirus epidemic, suffer from lung contamination. Early COVID-19 illness detection was achieved using imaging techniques, such as chest X-rays (CXR) and computed tomography (CT). Despite the fact that chest CTs have been shown to be a powerful imaging method for diagnosing lung-related illnesses, chest X-rays are more widely available because the diagnostic process is relatively quick [14].

#### 1.2.1. X-ray Radiography

X-rays, discovered in 1895 [15], are a sort of electromagnetic radiation. Medical X-rays are used to provide images of internal organs and tissues. These images depict body parts in various shades of black and white. CXR can be used for diagnosing bone fractures, some tumors, and diseases, such as COVID-19 [16]. Specifically, in COVID-19 detection, X-rays are thought to be one of the most effective methods.

#### 1.2.2. Computed Tomography

Another medical imaging technique, invented by South African scientist Allan Cormack, is computed tomography (CT), and is sometimes referred to as a CT scan [17]. CT is used in medical diagnosis to slice the imaging of different parts of the body and tissues, such as lungs, bones, veins, etc. Computed tomography plays an important role in the diagnosis of cancer [18], cardiovascular pathologies [19], trauma, and, more recently, COVID pneumonia [20].

### 1.3. Paper Structure

This paper provides a summary of various deep learning algorithms utilized in COVID-19 detection and classification using CT and X-ray radiography. The remainder of this review is organized in the following manner: in Section 2, basic and background information of deep learning techniques are presented. Deep learning systems for different image taxonomies are discussed in Section 3. Section 4 presents future directions and challenges. Finally, Section 5 provides the conclusion of this paper.

## 2. Basic and Background

Many studies have proposed different methods for separating COVID-19 pneumonia patients from healthy people. As of late, deep learning, a subset of machine learning [21], has exploded in popularity in the context of medical imaging analysis [22]. COVID-19 detection methods based on deep learning (DL) are being developed using CT and X-ray images [14,23,24,25]. Thus, DL techniques are regularly utilized to automatically extract features to classify cases infected with COVID-19. Components of these systems are built using a pre-trained model that incorporates transfer learning [26,27,28], and a few are introduced through personalized networks [29,30,31].

### 2.1. Deep Learning

**Figure 1:**
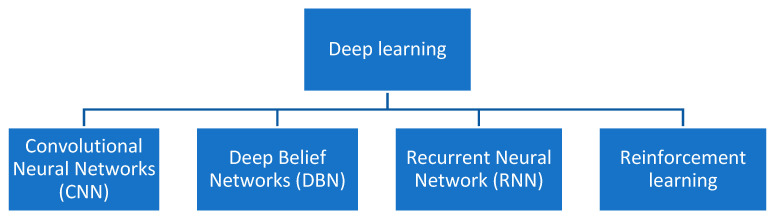
Deep learning-based COVID-19 diagnosis systems.

Deep learning, a machine learning subfield [32], is based on a network of artificial neurons inspired by the human brain [33]. The network is composed of several layers of neurons; each layer receives and interprets information from the previous layer. Deep learning models have had success in diagnosing system diseases. The convolutional neural network (CNN), recurrent neural network (RNN), deep belief network (DBN), and reinforcement learning are the four most used deep learning architectures (Figure 1).

### 2.2. Deep Learning Architectures

#### 2.2.1. Convolutional Neural Networks

**Figure 2:**
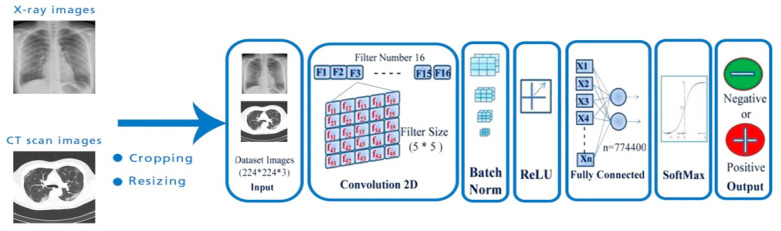
Architecture of a convolutional neural network (CNN) that helps to perform clinical diagnoses using X-ray and CT images.

Convolutional neural networks (CNN) are a particular type of multilayer perceptron [34], and have demonstrated outstanding performance in computer vision applications, such as image classification. The convolutional neural networks architecture is composed of a convolutional layer, pooling layer, and fully connected layer (see Figure 2). The convolutional layer plays a significant role in the CNN model. Using different types of filters (kernels), convolution extracts different features from an image, such as edges, textures, objects, and greater numbers of filters are used for the convolution process; an activation map is then generated to be fed as the input to the next layer of the CNN [35].

A pooling layer is incorporated between two convolutional layers and is used to reduce the size of images after they have been convoluted. There are three functions of pooling: max pooling, sum pooling, and average pooling. When applying a fully connected layer after two subsequent convolutional layers, without using average, max, or sum pooling, the calculations and parameter amounts are quite large [36,37].

The fully connected layer is also known as a dense layer and is used to identify an image with a probability value. After flattening, the output of the final pooling or convolutional layer becomes the input for the fully connected layer.

#### 2.2.2. Recurrent Neural Network

A sort of neural network known as a recurrent neural network (RNN) is a type of neural network with recurrent connections that uses sequential data or time-series data. It is used for pattern recognition of stream or sequential data, such as speech, handwriting, and text [38].

#### 2.2.3. Deep Belief Networks

Deep belief networks (DBNs) are probabilistic generative models with numerous layers of hidden variables. They are an effective method to resolve problems from neural network with deep layers, such as a low velocity and over fitting in learning. A DBN can be considered as the combination of a stack of restricted Boltzmann machines [39]. The layers of the RBMs are connected with previous and subsequent layers. Deep belief networks have two major characteristics: (1) learning top–down, and there is an effective layer-by-layer technique. The generative weights govern how variables in one layer interact with variables in another layer. (2) After learning, a single bottom–up run that starts with an observed data vector in the bottom layer and reverses the generating weights can infer the values of the latent variables in each layer [40].

#### 2.2.4. Reinforcement Learning

Reinforcement learning is a type of machine learning that recognizes and solves goal-oriented learning and decision-making problems automatically. Reinforcement learning is the set of methods that allows an agent to learn to choose, in an autonomous way, which action to take. It has been used in a variety of industries in recent years, with impressive results [41].

### 2.3. Transfer Learning

Transfer learning is a technique for reusing weights from a model that has been pre-trained on a larger dataset. Only the last few layers of the pre-trained model are replaced and retrained. Transfer learning is the process of leveraging a pre-trained model’s expertise to learn a new set of data [22]. This involves training CNNs using a large dataset to extract significant characteristics, and transferring this knowledge to re-train another CNN. There are several pre-trained models utilized in transfer learning, such as ResNet, AlexNet, GoogleNet, Visual Geometry Group (VGG), SqueezeNet, Inception, Xception, U-Net, MobileNet, DenseNet, etc.

### 2.4. Datasets

In Table 1, a summary of the publicly available datasets is presented. In deep learning applications, result accuracy depends on high-quality datasets. Several studies have integrated multiple datasets and used them with DL models to achieve enhanced performances in the detection of COVID-19. Both X-ray and CT images were obtained from public repositories, such as Kaggle and GitHub. Generally, two techniques are used for data partitioning: cross validation and splitting data into training, validation, and testing sets. COVID-19-detection systems based on deep learning have been created to serve as an accurate diagnosis for binary and multi classifications. Binary classification is a type of classification with a Boolean outcome (COVID or normal). Multi classification is a kind of classification where the output can be more than two values (COVID, normal, non-COVID viral pneumonia, non-COVID bacterial pneumonia, etc.).

### 2.5. Metrics

The assessment measures that are used to assess the performance of DL models are outlined in this section. Several benchmark metrics are utilized to evaluate the classification results. A number of metrics, including accuracy, sensitivity, specificity, recall, positive predictive value (PPV), precision, F1 measure (F1), area under the receiver operating characteristic curve (AUC), kappa criteria (Kappa), error, IoU, false positive rate, TNR, NPV, FPR, NPR, LRP, and LRN, are based on a confusion matrix (Table 2).

Table 3 summarizes a number of metrics used to evaluate the performance of deep learning systems developed for the detection and classification of COVID-19.

## 3. Deep Learning Techniques for Different Image Modalities

In this paper, we present 50 papers covering COVID-19 classification methods. Twenty-one techniques (42% of the total number of reviewed systems) deal with binary classification and 29 (68% of the total reviewed number of systems) deal with multi-class classification.

Before proceeding with the classification step, the preprocessing phase needs to be underlined. In fact, for AI-based COVID-19 image processing and analysis, segmentation is a crucial stage. It delineates regions of interest (ROIs) in chest X-rays or CT images, such as the lung, lobes, bronchopulmonary segments, and infected regions or lesions, for further assessment and quantification.

We summarize the literature-based information on COVID-19 infection segmentation processes presented in the most recent studies (Table 4).

For image tissue classification, the authors of [42] proposed using two well-known deep learning networks, SegNet and U-NET. U-NET is a medical segmentation tool, while SegNet is a scene segmentation network. Both networks were used as binary segmentors to distinguish infected from healthy lung tissues, as well as multi-class segmentors to learn the type of infection in the lung. The obtained results demonstrated that SegNet outperformed the other approaches in classifying infected/non-infected tissues (with a 0.95 mean accuracy), while U-NET outperformed the others as a multi-class segmentor (with a 0.91 mean accuracy).

Using 929 lung CT images, the authors of [43] proposed a novel segmentation approach named AnamNet. Compared to the state-of-the-art UNet, the proposed Anam-Net had 7.8 times fewer parameters (or variants). The results demonstrated that the suggested method provided good Dice similarity scores for diseased and normal lung regions, with an accuracy of 98%. In [44], the authors designed an encoder–decoder segmentation approach called SD-UNet. The metrics of sensitivity, accuracy, specificity, and similarity were 0.8988, 0.8696, 0.9906, and 0.7702, respectively.

Shan et al. suggested a DL-based segmentation method (VB-Net) for segmenting COVID-19 infection areas in CT scans, which was tested on a dataset of 249 images [45]. It took the form of a 3D convolutional neural network with a bottleneck structure that combines V-Net and a bottleneck structure. VB-Net has two methods to extract global image features: the first approach is to contract a path that includes down-sampling and convolution procedures. The second path is a broad one, which incorporates fine-grained image features through up-sampling and convolution processes.

In [46], it was suggested that a CNN model could be used for COVID-19 lung CT segmentation (SSInfNet). The self-supervised InfNet incorporated various techniques, such as generative adversarial image inpainting, lookahead optimizer, and focal loss. The used dataset consisted of 7586 CT samples, 698 that were used for training, 6654 for validation, and 117 that were used for testing the system. SInfNet achieved an F1 score, recall, and precision of 63%, 71%, and 68% respectively.

COVLIAS 1.0 is a COVID lung image analysis system that was proposed in [47]. The system is composed of three methods SegNet, VGG-SegNet, and ResNet-SegNet. Using a dataset of 5000 lung CT images, COVLIAS 1.0 was benchmarked against the NIH (National Institute of Health) and was founded on a conventional segmentation model using fuzzy-connectedness. The obtained results demonstrated that the three models were better than the conventional NIH model.

Based on the encoder–decoder architecture, a novel segmentation technique was proposed in [48], built on combining multi-scale feature maps of multiple levels. The suggested schemes were validated using four different COVID-19 CT datasets. The results revealed that all three of the proposed modules, the edge supervised module (ESM), semantic supervised module (ASSM), and attention fusion module (AFM) with ResUnet improved the Dice metric by 3.97%.

Segmentors are also considered to be classifiers. As mentioned above, we studied 50 works on deep learning using different images modalities. For each type of classification, the deep learning methods were divided into two categories: pre-trained models with deep transfer learning and personalized deep learning techniques.

### 3.1. Binary Classification

The binary classification is the sort of classification where the output is two classes: COVID-19 or normal, COVID-19 or non-COVID-19, and COVID-19 or pneumonia.

#### 3.1.1. Pre-Trained Model with Deep Transfer Learning

In [49], the authors suggested a deep learning architecture for detecting COVID-19 illnesses using X-ray and CT scan pictures of the chest. For diagnoses, the system utilized VGG16, VGG19, Xception, ResNet50V2, MobileNetV2, NasNetMobile, ResNet101V2, and InceptionV3 CNN architectural versions. A total of 1000 X-ray and CT scans were used in the investigation, with 805 images from healthy people and the rest from COVID-19 patients. The dataset was divided into two parts, with 80% of the data used for training and 20% used for testing. The VGG-19 model had the best accuracy, with a score of 99%.

In [50], two in-depth learning techniques, SegNet and U-NET, were proposed to semantically segment infected tissue regions in CT lung images. Both networks were utilized as binary segmentors to distinguish between healthy and infected lung tissues, and multi-class segmentors were used to determine what type of lung infection is present. The experimental findings reveal that SegNet performed comparatively better than the other method in classifying infected/non-infected tissues (with 95% mean accuracy). U-NET obtained better results as a multi-class segmentor (with 91% mean accuracy).

In [50], a method based on deep learning networks was introduced to diagnose COVID-19 based on X-ray images using a pre-trained model (ResNet50). The dataset contained 50 X-ray images for lungs, where 25 X-ray images were for patients with COVID-19 and 25 X-ray images were for healthy patients. For the experiment, 5- and 10-fold cross validation used to split the dataset. The model achieved an accuracy of 97.28% in 5-fold cross-validation experiments and 95.99% in 10-fold cross validation experiments.

In [51], a deep learning-based system for detecting, localizing, and quantifying COVID-19 manifestation severity from chest CT scans was suggested. Using 1865 CT images, the model was trained and tested. The system had an AUC of 99.4%, a sensitivity of 94%, and a specificity of 98%, according to the results of the trial.

Table 5 summarizes deep learning models for binary classification of COVID-19 utilizing a pre-trained model and deep transfer learning. Different medical imaging modalities were used in the DL techniques, including computer tomography (CT) and chest X-rays (CXR).

#### 3.1.2. Custom Deep Learning Techniques

In [57], a deep learning model with stochastic pooling for COVID-19 detection was proposed. The system considered 640 CT images from two classes, where 320 samples were COVID-19 cases and 320 were healthy samples. To obtain a better performance, the collected dataset was divided using the 10-fold cross-validation method. The proposed system found a sensitivity of 93.28% ± 1.50%, specificity of 94.00% ± 1.56%, and an accuracy of 93.64% ±1.42%. In another study, the authors of [58] presented a custom-designed architecture with optimized parameters of variants of a convolutional neural network (CNN). In this work, the system used 753 X-ray images, in which 253 were tagged as COVID-19 and 500 were tagged as normal. Five-fold cross validation was used to test the suggested model. The dataset was split into two sections: training (653 X-ray images) and hold out (653 X-ray images) (100 X-ray). The training set was divided 5-fold, while the hold out part was aimed at testing the model at the end. The experimental results achieved a precision of 99%, recall of 99%, F1 score of 99%, AUC of 99%, and MCC of 99%.

In another research work [59], a diagnosis prototype system based on ResNet50 architecture was proposed. The used COVID-19 CT dataset of the study was obtained from Huangpi Hospital of Traditional Chinese Medicine, Wuhan, China. In this experiment, 1867 CT samples were used for training, 1400 CT samples were used for validation, and 510 samples were used for testing. The experimental results showed that the system obtained an accuracy of 93%, sensitivity of 93%, specificity of 92%, F1 score of 92%, IoU of 85%, and AUC of 93%. In [60], an intelligent decision support system for COVID-19 powered by deep learning (ID2S-COVID19-DL) using X-ray and CT-scan images was presented. The dataset was collected from different sources, such as cameras, X-rays, and CT-scan machines through the Internet of Medical Things (IoMT). The dataset was divided into two sets: training and validation, with each set accounting for 80% and 20% of the total, respectively. The created system had a 95.5% accuracy rate.

Recently, in [61], a new neural network was built for detecting COVID-19 from CXR images that blends topological and deep characteristics (TDA-Net). TDA-Net has two branches: a deep branch that accepts a raw image and a topological branch that accepts a topological feature vector. Both branch outputs are then combined and used to perform a classification. The data were collected from two open-source datasets of chest X-ray and CT images [53,54,55,56,57,58,59,60,61,62]. The first dataset consisted of 351 chest X-ray and CT images, which were positive or suspected of COVID-19. The second dataset from Kaggle contained 112,120 X-ray images (287 samples of chest X-ray images of viral and bacterial pneumonia were selected). The data were divided into two parts. The test set comprised 20% of the overall dataset, with 116 samples being evenly distributed between the positive and negative classes. The suggested that the network had a 93% accuracy rate.

The authors in [63] introduced a deep learning algorithm based on a modified CNN. In the experiment, a total of 1065 CT images were used for the training set, 455 images were used for the internal validation, and the rest were used for external validation. The external testing dataset achieved a total accuracy of 79.3%. The authors of [64] defined a fully automated system for COVID-19 detection from CT scans. The proposed system made use of the ResNet50V2 model, which is a popular pre-trained model with a feature pyramid network (FPN). In the study, they introduced a new dataset named COVID-CTset. Among the 63,849 images, 15,589 were confirmed COVID-19 cases and 48,260 were normal cases. The scheme used 5-fold cross validation for data partitioning. The system obtained an accuracy of 98.49%. In [65], pre-trained CNN and J48 models were used to construct a system for detecting COVID-19. To extract the features, the algorithm used eleven different architectures of pre-trained models (AlexNet, VGG16, VGG19, GoogleNet, ResNet18, ResNet50, ResNet101, InceptionV3, InceptionResNetV2, DenseNet201, and XceptionNet), as well as J48 for COVID-19 chest X-ray image classification into normal and COVID-19 cases. With accuracy, recall, specificity, precision, and F1 scores of 100 percent, 100 percent, 98.89%, 100%, and 100%, respectively, the Resnet101 and J48-based CNN methods were superior for the detection of COVID-19.

To detect pneumonia, the authors of [66] created the CGNet framework, a novel deep learning model. The dataset was collected from two public datasets. The proposed system achieved an accuracy of 98.72% on a public pneumonia dataset, which included 5856 chest X-ray images. The proposed technique was evaluated on a public COVID-19 CT dataset for the detection of COVID-19 pneumonia. The system achieved an accuracy of 99%, specificity of 100% and sensitivity of 98%, respectively. In [67], an ensemble of convolutional neural networks was developed to detect COVID-19 and was named DeepCOVID-XR. The proposed algorithm was trained and validated on 13,156 CXR images and then tested on 1879 CXR images. For the entire test, DeepCOVID-XR obtained an accuracy of 83%, and an AUC of 90%. For 300 random test images, the system achieved an accuracy of 82%. The authors of [68] described a powerful deep learning strategy for detecting coronavirus infection. Convolutional neural networks (CNN) and convolutional long short-term memory (CLSM) were used in the suggested system (ConvLSTM). The network was tested on both CT and X-ray images, and on a combined dataset (X-ray and CT). To achieve a better result, the dataset was divided into 70% training and 30% testing sets. In other circumstances, the proposed CNN modality obtained a 100% accuracy and a 100% F1 score.

The authors of Saha [69] advocated using X-ray images to identify COVID-19 patients using an automated detection system called EMCNet. EMCNet uses CNN to extract features from images and an ensemble of four different ML classifiers to classify COVID-19 (random forest, support vector machine, decision tree, and AdaBoost). The dataset was divided into three parts: training, validation, and testing. The training, validation, and testing sets each received 70%, 20%, and 10% of the total set of images. EMCNet obtained accuracy, precision, recall, and F1 score of 98.91%, 100%, 97.82%, and 98.89%, respectively.

In [70], pre-trained CNN models were used to construct an autonomous approach for diagnosing coronavirus from CT images. The proposed system combined two variants of CNNs (ResNet5 and ResNet-101). ResNet50 was utilized to distinguish virally induced pneumonia from bacterially induced pneumonia and normal cases in this investigation, while ResNet-101 was used to detect the presence of COVID-19 in positive viral-induced pneumonia patients using X-ray images. The data were collected from two open-source image databases, Cohen and Kaggle. Among the 1365 chest X-ray images, 250 were confirmed as COVID-19. To obtain better performance, two evaluations were used: training–validation–testing and 5-fold cross validation procedures. The developed system obtained a high classification accuracy of 97.77%. Further, the proposed model achieved an averaged accuracy with 5-fold cross validation.

In [71], the authors described a COVID MTNet system for COVID-19 identification and contaminated region localization using two medical imaging modalities (X-ray and CT images). The inception recurrent residual neural network (IRRCNN) and NABLA-3 network models were used in the study for the classification and segmentation tasks. There were a total of 5216 samples, with only 1341 samples for normal cases and 3875 samples for pneumonia. The created system had an X-ray image testing accuracy of 84.67% and a CT image testing accuracy of 98.78%. In a different project [72], 3D CT volumes were used to construct a weakly-supervised deep learning-based software solution to detect COVID-19 (DeCoVNet). A pre-trained UNet was used to segment the lung region, and the segmented 3D lung region was then fed into a 3D deep neural network to predict the likelihood of COVID-19 being infectious. The data were split into two parts: training (499 CT volumes) and testing (499 CT volumes) (131 CT volumes). The proposed system had a ROC AUC of 95.9% and a PR AUC of 97.6%, respectively.

In [73], a system for diagnosing coronavirus from CT images was suggested, based on a deep learning algorithm called CTnet-10, which is a variation of CNN. This study used 738 CT scan pictures, 349 of which were obtained from COVID-19-infected patients and 463 were from non-COVID-19-infected patients. The data were divided into three sets: training, validation, and test, in a ratio of 80:10:10. The designed system achieved an accuracy of 82.1% in the test case.

Table 6 presents a summary of the deep learning models used for binary classification of COVID-19 using custom deep learning techniques. The DL methods employed different medical imaging modalities: computer tomography (CT) and chest X-rays (CXR).

### 3.2. Multi-Classification

#### 3.2.1. Pre-Trained Model with Deep Transfer Learning

The authors of [83] developed a COVID-19 detection framework that used the notion of a pre-trained model to automatically classify positive COVID-19 chest X-rays and CT scans into three severity classes: normal, mild/moderate, and severe. The suggested approach combined transfer learning with three prominent pre-trained CNN models: AlexNet, GoogleNet, and Resnet50. The system considered 1491 chest X-rays and CT scans, including 1335 normal, 106 mild/moderate, and 50 severe cases for experiments. The dataset was divided into three parts, 70% for training, 15% for validation and 15% for testing. ResNet50 outperformed the other models used and obtained an overall accuracy of 87.8%.

The authors of [84] suggested a three-label classification framework with an ensemble of convolutional neural network (DenseNet161) models concentrating on both global and local pathological variables from CXR lung images to detect COVID-19. In this system, 11,197 CXR images were considered, 1056 samples were COVID-19, 5451 were pneumonia, 931 were viral pneumonia, and 7217 were control (normal and other pulmonary diseases). The split of the dataset was 70%, 15%, and 15% for training, validation, and testing, respectively. In a multi-label classification framework that included COVID-19, pneumonia, and control classes, the suggested system achieved an average balanced accuracy of 91.2%, average precision of 92.4%, and F1 score of 91.9%.

In another research project [85], DenseNet-121 was used to construct a deep learning-based strategy for detecting COVID-19 patients. The suggested system was trained and tested using the COVIDx dataset, which included 13,800 chest radiography pictures from 13,725 patients. To get a better result, the obtained dataset was divided using the 10-fold cross-validation approach. The model was put to the test for two-class classification (COVID-19 and non-COVID-19) and three-class classification (COVID-19 and non-COVID-19) (COVID-19, pneumonia, and normal). The proposed network achieved a 96.49% accuracy for the two-class classification and 93.71% accuracy for the three-class classification. In [86], a framework of cascaded deep learning classifiers for automated diagnosis of COVID-19 and pneumonia diseases using chest X-rays was proposed. VGG16, VGG19, Xception, dense convolutional network (DenseNet-121), DenseNet169, DenseNet201, residual neural network (ResNet-50V2), ResNet101V2, ResNet169V2, MobileNet, and MobileNetV2 are some of the deep learning models used in this architecture. VGG16, ResNet50V2, and dense neural network (DenseNet169) were the top fine-tuning models in terms of detection accuracy (99.9 percent). For identifying COVID-19 chest X-ray images, the authors of [87] used a light-weight convolutional network architecture with three backbones (VGG-16, ResNet50, and EfficientNetB0). In this research, the dataset was collected from two available chest X-ray datasets. The datasets maintained a ratio of 80% and 20% for training and testing sets, respectively. The proposed models achieved an overall accuracy of 90%, 94.3%, and 96.8% for VGG16, ResNet50, and EfficientNetB0 backbones, respectively.

In [88], CXR images were used to build a technique for detecting COVID-19 pneumonia, non-COVID-19 viral pneumonia, bacterial pneumonia, and healthy patients. AlexNet was the pre-trained model in this system. The datasets were separated into two categories: 70% for training and 30% for testing. The network was trained to perform two-way classification, three-way classification, and four-way classification (COVID-19 vs. normal, bacterial pneumonia vs. normal, non-COVID-19 viral pneumonia vs. normal, and COVID-19 vs. bacterial pneumonia). The model achieved a 99.62% testing accuracy, 90.63% sensitivity, and 99.89% specificity for the classification of COVID-19 pneumonia and non-COVID-19 viral pneumonia.

In [89], a COVID-19 detection model based on Inception V3, Xception, and ResNeXt architectures was suggested. A total of 6432 CXR scan samples were acquired from a Kaggle library for the research. A total of 5467 samples were utilized for training, while 965 samples were used for validation. In comparison to other models, the Xception model fared better. For detecting chest X-ray pictures, Xception had an overall accuracy of 97.97%. The authors [90], also described a method that uses transfer learning and model integration to detect COVID-19. The information was gathered from two different datasets: the RSNA pneumonia dataset and the chest X-Ray dataset. The dataset was split into two sections: training (16,714 X-ray images) and testing (16,714 X-ray images) (1862 X-ray). On the testing set, the suggested model correctly identified 96.1% of the types of chest X-ray images.

In [91], a method for detecting coronavirus illness based on deep transfer learning and several pre-trained models was proposed. VGG16, VGG19, DenseNet201, Inception ResNet V2, Inception V3, Resnet50, and MobileNet V2 are the seven most common pre-trained models. For the experiments, 6087 chest X-ray images and CT images were used (2780 images of bacterial pneumonia, 1493 images of coronavirus, 231 images of COVID-19, and 1583 normal images). In this system, the training and validation data partitions were kept at an 80:20 ratio. Densnet201 and Inception Resnet V2 performed better than the other models employed in the study (92.18% accuracy for Inception-ResNetV2 and 88.09% accuracy for Densnet201).

Table 7 summarizes the deep learning models for multi-class classification utilizing a pre-trained model with deep transfer learning for the COVID-19 dataset. Different medical imaging modalities were used in the DL techniques (computer tomography (CT) and chest X-rays (CXR)).

#### 3.2.2. Custom Deep Learning Techniques

The work in [99] introduced an ensemble deep learning model for novel COVID-19 detection from CT images. The ensemble classifier, EDL-COVID, is based on three deep convolutional neural network models: AlexNet, GoogleNet, and ResNet. The used dataset consisted of 2500 CT images of lung tumors and 2500 normal lungs. The proposed model was evaluated using 5-fold cross validation. EDL-COVID obtained an accuracy, sensitivity, specificity, F-measure, and MCC of 99.054%, 99.05%, 99.6%, 98.59%, and 97.89%, respectively.

Authors of another study suggested a deep learning diagnostic assistance system for COVID-19 detection using chest radiographs [100]. The system employed a modified and expanded version of COVID-deep net’s learning algorithm. Five open-access databases were used to compile the data. Following data harmonization, the training set included 7966 normal cases, 5451 with other pneumonia, and 258 CXRs with COVID-19 pneumonia, where each group was represented by 100 cases in the testing dataset. The overall diagnostic accuracy for the suggested approach was 94.3%.

To distinguish the infected cases from the normal or pneumonia cases, other authors [13] used the modified ResNet18-based convolution neural networks with chest X-ray images. In this system, 15,085 X-ray images were used for the diagnosis. The dataset was split using 3-fold cross validation. The proposed model obtained an accuracy of 96.73%, recall of 94%, and specificity of 100% for the three classes (normal, pneumonia, and COVID-19). In another study [101], a computer aided diagnostic (CAD) framework comprised of two deep learning models (discrimination-DL and localization-DL) were proposed. The used dataset consisted in 3545 chest X-ray samples where 204 samples were COVID-19 cases, 2004 samples were CAP cases, and 1314 samples were healthy people. To obtain a better performance, the dataset was divided into a 80% for training and 20% for validation, and 61 images were collected from 21 COVID-19 patients, 20 CAP patients, and 20 controls, which were used in the testing phase to prove the model generalization. The final CAD scheme achieved a test accuracy of 93.65%, sensitivity of 90.92%, and specificity of 92.62%.

The authors of [102] introduced a deep learning approach (CNN with five convolutional layers) for COVID-19 and viral pneumonia screening using X-ray images. In the study, X-ray images were collected from Kaggle [53,92]. The used dataset contained 1389 images. The proposed deep learning model produced an average classification accuracy of 90.64% and an F1 score of 89.8% after performing 5-fold cross validation on a multi-class dataset consisting of COVID-19, viral pneumonia, and normal X-ray images.

The authors of [103] described two deep learning architectures for automatically detecting COVID-19-positive patients using chest CT X-ray pictures. The modified AlexNet (mAlexNet) architecture was the first proposed architecture. AlexNet is made up of 25 layers, one of which is a convolution layer. Bidirectional long short-term memories (BiLSTM) is the second architecture. A total of 2905 chest X-ray images were used in the study. The authors employed a variety of indicators to assess their proposed models. With a 98.70% accuracy, BiLSTM outperformed AlexNet.

The authors of [104] suggested an integrated stacked deep convolution network, InstaCovNet-19. To compensate for the small size of training dataset, the created system utilized different pre-trained models, ResNet101, Xception, InceptionV3, MobileNet, and NASNet. The suggested approach used X-ray images of a sick person’s chest to detect COVID-19 and pneumonia. There were 361 verified COVID-19 instances, 1341 pneumonia cases, and 1345 normal cases among the 3047 chest X-rays. The dataset was partitioned into a training and testing set a a ratio of 80% and 20%, respectively. The proposed model achieved an accuracy of 99.08% for the three classes (COVID-19, pneumonia, and normal), while achieving an accuracy of 99.53% for two classes (COVID-19, healthy). The proposed system achieved an average recall, F1 score, and precision of 99%, 99%, and 99%, respectively, for multi classification, while achieving a 100% precision and a recall of 99% for the binary classification.

The authors of [105] used shuffled residual CNN to determine different filters for COVID-19 detection from chest X-rays. The proposed work included two CNN architectures: channel-shuffled dual-branched (CSDB) CNN and CSDB CNN with a distinctive filter learning (DFL) paradigm. In the study, a total of 3047 chest X-ray images were taken, where 10,434 were from healthy people (normal), 558 were COVID-19 cases, 2780 were bacterial pneumonia cases, and 1493 cases were viral pneumonia diseases. In this scheme, the dataset was partitioned using a 5-fold cross-validation technique. The proposed system (customized CNN with a distinctive filter learning module) obtained an F1 score of 97.20% and an accuracy of 99.80% for the COVID-19 X-ray set.

The authors of [106] proposed binary and multi-classification deep learning models. The acquired data were divided into two sets: training and testing, at 80% and 20% respectively. The binary model had a precision of 98.7%, while the three-class model had an accuracy of 98.3%.

The author of [107] described an MH-COVIDNet system that used deep neural networks and meta-heuristic-based feature selection on X-ray images to diagnose COVID-19. A dataset of 364 X-ray images of COVID-19, normal, and pneumonia, was constructed for this investigation, with each class having 364 images. The 5-fold cross-validation approach was used to partition the dataset. The accuracy of MH-COVIDNet was 99.38%.

In another research work [108], a novel CNN model called CoroDet was introduced for the automatic detection of COVID-19 using raw chest X-ray and CT scan images. CoroDet was developed to serve as an accurate diagnostic for binary and multi-classes. A total of 7390 images were considered for the experiment. The dataset was divided using the 5-fold cross-validation method. The twenty-two-layer CNN model achieved an accuracy of 99.1% for binary classification, 94.2% for three classes, and 91.2% for four classes.

In [24], COVIDCTNet, an open-source deep learning technique for diagnosing COVID-19 based on a small cohort of CT images was suggested. In the CNN evaluation, the dataset was split at 95% for the training the algorithm and 5% for validating the model in the hold-out. During the validation phase, the suggested system achieved a detection accuracy of 93.33% of COVID-19 versus non-COVID-19 (two classes) and a multi-classification accuracy of 86.66% was achieved. To test the classification quality of the model, an independent dataset consisting of 20 mixed cases of control, COVID-19, and CAP was used. COVIDCTNet achieved an accuracy of 95% for two classes (COVID-19 cases, non- COVID-19) and an accuracy of 85% for three classes.

In another work [109] a novel COVID-19-assisted diagnosis schema, based on a convolution neural network, was proposed. The COVID-19 dataset was composed of 1184 X-ray images of COVID-19, MERS SARS, ARDS illnesses, and normal cases. All of the data were divided into two categories: training (757 images) and testing (427 images). The network obtained an accuracy, precision, recall, and F1 score of 98%, 99%, 98%, and 98%, respectively.

In [110], the Convid-Net deep convolutional neural network (CNN) framework for detecting COVID-19 from chest X-ray pictures, which was based on a combination of a residual network and parallel convolution. In the work, the dataset was retrieved from different publicly available sources, consisting of a total of 1440 COVID-19 images, 2470 normal images, and 2407 chest X-ray images of viral and bacterial pneumonia. Convid-Net achieved an accuracy of 97.99%. The authors of [111] suggested a lightweight deep convolutional neural network for chest X-rays. The proposed architecture was inspired by InceptionV3, InceptionResNetV2, and MobileNetV2. The dataset was collected from three different open access datasets. The used data were partitioned into 20,907 training samples and 231 testing samples. The proposed model achieved a 95% accuracy for multi-classification.

DeepCoroNet, a method based on a deep LSTM model for automatically identifying COVID-19 instances from X-ray pictures, was introduced in [112]. To execute the experiment, different ratios of training and testing datasets (60:40%, 70:30%, and 80:20%) were used. The best results were obtained with an 80% training rate and a 20% testing rate. All performance criteria were met by the network, which included accuracy, sensitivity, specificity, and F score.

In another study, the authors of [113] established a deep learning framework for detecting COVID-19 in X-ray and computed tomography images. ResBlock-A, ResBlock-B, and Control Gate Block made up a modular CNN-based classification system. The data for the study were gathered from a variety of sources. The suggested system used 9830 images for training and 547 images for testing from the total dataset. The trial results yielded an F1 score of 98.90% and a specificity of 100%.

In [114], COVID-19 infected cases from four other classes, normal, tuberculosis (TB), bacterial pneumonia (BP), and viral pneumonia (VP), were classified using a deep learning technique CNN named MANet. The proposed system contained a two-stage segmentation using the UNet model with a ResNet backbone and classification was performed by including four classic CNNs (ResNet34, ResNet50, VGG16, and Inceptionv3). The datasets were collected from three public CXR data repositories, and consisted of CXR images from five classes, normal, COVID-19, TB, BP, and VP with 1840, 433, 394, 2780, and 1345 images, respectively. ResNet50 with MA scored the highest average test accuracy of 96.32% in three runs, and the highest one was 97.06%, among the tested classification models.

The authors of [115] presented COVID-19 detection utilizing deep learning models and structured chest X-ray images using fuzzy color and stacking algorithms to exploit social mimic optimization. In the study, the dataset consisted of three classes; namely, coronavirus, pneumonia, and normal X-ray imagery. In preprocessing, the dataset was reconstructed using the fuzzy technique and the stacking technique. The MobileNetV2 and SqueezeNet deep learning models were trained using the stacked dataset. The obtained feature sets were classified using the SVM method. The dataset was split up into 70% and 30% for the training and testing sets, respectively. For the experimentation related to the stacked dataset, the k-fold cross-validation method was used. The proposed approach achieved an overall accuracy of 99.27%.

Using chest X-rays, in [116], a confidence-aware anomaly detection (CAAD) model was developed to differentiate viral pneumonia cases from non-viral pneumonia cases and healthy controls. The X-VIRAL and XCOVID X-ray image collections were used in this work. There were 5977 instances of viral pneumonia, 18,619 cases of non-viral pneumonia, and 18,774 healthy controls in the X-VIRAL dataset (5977 positive and 37,393 negative cases). A total of 106 verified COVID-19 cases and 107 healthy controls made up the X-COVID set. For external validation, a public COVID-19 dataset called Open-COVID was employed. The X-ray images of 493 confirmed COVID-19 patients, 16 confirmed SARS cases, and 10 confirmed MERS cases were included in the dataset. During testing, the proposed design achieved an AUC of 83.61% and had a sensitivity of 71.70%.

CVDNet is a unique deep learning architecture created by the authors of [117] for identification of coronavirus (COVID-19) from chest X-ray images. The convolutional neural network (CNN) model was trained on a dataset that included 219 COVID-19, 1341 normal, and 1345 viral pneumonia chest X-ray images, and which is publicly available. The dataset was separated into three classes using the 5-fold cross-validation procedure. To classify COVID-19, normal, and viral pneumonia, the proposed model had an average accuracy of 97.20%.

Table 8 summarizes the deep learning models for multi-class classification utilizing a pre-trained model with deep transfer learning for the COVID-19 dataset. Different medical imaging modalities were used in the DL techniques (computer tomography (CT) and chest X-rays (CXR)).

## 4. Discussion: Challenge and Future Research Direction

This section provides some directions can be utilized in future research in the detection and classification of coronavirus and enhance the efficiency of future deep learning classifiers. Some challenges were inspired by [131].

To begin, it is vital to emphasize that some studies examined, analyzed, and evaluated distinct datasets that were privately obtained by clinics, hospitals, or COVID research institutes. The main drawbacks of this are that it is difficult to go against the performance of these models in different studies.

Additionally, the training process plays an essential role in deep learning; to have a good model, huge amounts of training data are needed. At the start of the pandemic, the lack of datasets for training deep learning models for medical imaging (CT or X-rays) was a major challenge. In general, collecting and labeling large amounts of medical imaging data is difficult because it requires a great deal of time and effort by radiologists (experts). Several factors can be involved in collecting data, such as lighting conditions, different presentation characteristics of coloring, various sizes and views in different image modalities, and enlargement. It is important to consider the influence of clinical situations and collection techniques on the robustness of a dataset.

In the reviewed COVID-19 applications, authors used the classification of COVID-19 based on the supervised learning method. With this approach, training the models with tagged images led to better results. From the beginning of December 2019, the outbreak of COVID-19 has put health care systems under tremendous pressure. Thus, it is difficult to gather images of correct indications of COVID-19 that have been labeled by professional doctors. Generally, there are a number of unidentified clinical images that are accessible. These unlabeled images are a major source of knowledge and cannot be used for supervised learning. Hence, a classification model for COVID-19 is desperately needed and can be trained using several of clustering methods without supervision [132].

Another limitation in some studies is the use of data augmentation approaches rather than transfer learning to prevent over fitting. Most research studies applied data augmentation techniques, including translation, horizontal (and vertical) flipping, and random rotation to avoid the over fitting and to enhance the accuracy of model predictions [133]. Data augmentation is a good tool to solve the problems of unbalanced data or a lack of data; it can generate new images that retain the original features.

An additional concern is data leaking, which is one of the most serious and widespread issues in machine learning, as well as in deep learning. The most of the time, it can occur in the feature engineering stage in the pre-processing phase. Generally, this problem is caused by missing values, temporal data, and the normalization of data. In the context of training dataset using CT or CXR images, the normalization stage of the whole dataset [24] can be applied before splitting, and, at that time, a part of the information from the training and testing dataset can be shared. Unfortunately, during the splitting phase, there is no guarantee that all images from one patient will be placed into one sample set because all the samples are taken at random without any restriction. Data leakage can be avoided by properly performing cross validation.

Finally, the absence of benchmarks for COVID-19 classification systems based on deep learning was viewed as a challenge and resulted in an absence of flexibility.

Diagnosis and treatment of COVID-19 is essential. In the absence of a good cure, we just need to identify additional AI-based DL techniques for the early detection of COVID-19.

In order to prevent disease and the progression of the pandemic, it is necessary to detect and diagnose COVID-19 quickly using DL applications at the lowest cost and with few complications. The integration of DL techniques in radiology centers enables rapid and accurate diagnoses of pneumonia, especially in cases of COVID-19. The incorporation of DL methods in healthcare systems aids in decision making and a reduction in human error.

The majority of research on deep learning techniques, distinguishes COVID-19-infected cases from the other classes, such as normal, tuberculosis (TB), bacterial pneumonia (BP), and viral pneumonia (VP) cases.

The World Health Organization (WHO) designated certain Pango lineages as variations of concern (VOC) and assigned Greek letter designations, such as alpha (Pango lineage designation B.1.1.7), beta (B.1.351), delta (B.1.167.2), and, most recently, omicron (B.1.1.529). There are variations among these strains that are more communicable and others that are even more difficult to detect using traditional diagnostic techniques. Currently, there is a pressing need to create deep learning algorithms that can accurately and swiftly detect and classify the many SARS-CoV-2 mutations.

The reinforcement learning methodology allows a deep learning model to learn from its environment. The development of a system based on reinforcement learning can convincingly increase the efficiency and performance of COVID-19-classification techniques using different modalities of medical images.

## 5. Conclusions

In conclusion, the review focused on approaches based on deep learning networks for automated COVID-19 detection. The algorithms created in previous studies for the detection and classification of SARS-CoV-2, using deep learning approaches, with two imaging modalities (CT and X-ray samples), are described in this paper. Several studies have combined multiple datasets and used them in DL models to improve COVID-19-detection performance. In this paper, we collected sources of used datasets that can be easily accessed by researchers. The major challenge was absence of benchmarks for COVID-19 classification systems based on deep learning. We desperately need to develop deep learning systems with a higher performance in identifying COVID-19 at an early stage and that supports radiologists in their diagnoses.

## Figures and Tables

**Table 1:** Summary of publicly available datasets used in the relevant publications and corresponding URLs (accessed on 17 December 2021).

Databases	Sources (URL)
COVID-19 Image Data Collection	https://github.com/ieee8023/COVID-chestxray-dataset
COVID-19 Chest X-ray	https://github.com/agchung/Figure1-COVID-chestxray-dataset
ActualMed COVID-19 Chest X-ray dataset	https://github.com/agchung/Actualmed-COVID-chestxray-dataset
COVID-19 Radiography Database	https://www.kaggle.com/tawsifurrahman/COVID19-radiography-database
RSNA Pneumonia Detection Challenge dataset	https://www.kaggle.com/c/rsna-pneumonia-detection-challenge/data
COVID-19 X-ray images	https://www.kaggle.com/bachrr/COVID-chest-xray
COVID-19 detection X-ray dataset	https://kaggle.com/darshan1504/COVID19-detection-xray-dataset
NIH chest X-ray dataset	https://www.kaggle.com/nih-chest-xrays/data
COVID-CT	https://github.com/UCSD-AI4H/COVID-CT
Chest X-ray images (pneumonia)	https://www.kaggle.com/paultimothymooney/chest-xray-pneumonia/version/1
SARS-CoV-2 CT-scan dataset	https://www.kaggle.com/plameneduardo/sarscov2-ctscan-dataset
COVID-19 X-ray dataset (training and testing sets)	https://www.kaggle.com/khoongweihao/COVID19-xray-dataset-train-test-sets
COVID-CTset	https://github.com/mr7495/COVID-CTset
Chest X-ray (COVID-19 and pneumonia) COVID-19 COVID-19 CT Lung and Infection Segmentation dataset Labeled COVID-19 CT scans	https://www.kaggle.com/prashant268/chest-xray-COVID19-pneumonia https://mosmed.ai/en/ http://medicalsegmentation.com/COVID19/ https://gitee.com/junma11/COVID-19-CT-Seg-Benchmark

**Table 2:** Confusion matrix.

	Predicted Class	
**Actual class**	True Positive (**TP**)	False Positive (**FP**)
	False Negative (**FN**)	True Negative (**TN**)

**Table 3:** Summary of benchmarks metrics used in the relevant publications in this review.

Metrics	Definition
Accuracy	$\mathrm{Measure}\;\mathrm{indicates}\;\mathrm{the}\;\mathrm{percentage}\;\mathrm{of}\;\mathrm{correct}\;\mathrm{predictions},\;Accuracy=\frac{\left(\mathrm{TP}\;+\;\mathrm{TN}\right)}{\left(\mathrm{TP}\;+\;\mathrm{TN}\;+\;\mathrm{FP}\;+\;\mathrm{FN}\right)}$.
Precision/PPV	$\mathrm{Measure}\;\mathrm{indicates}\;\mathrm{the}\;\mathrm{percentage}\;\mathrm{of}\;\mathrm{correct}\;\mathrm{positive}\;\mathrm{predictions},\;Precision=\frac{\mathrm{TP}}{\left(\mathrm{TP}\;+\;\mathrm{FP}\right)}$.
Recall /Sensitivity/TPR	$\mathrm{Measure}\;\mathrm{indicates}\;\mathrm{the}\;\mathrm{percentage}\;\mathrm{of}\;\mathrm{positive}\;\mathrm{labeled}\;\mathrm{samples}\;\mathrm{that}\;\mathrm{were}\;\mathrm{predicted}\;\mathrm{as}\;\mathrm{positive},\;Recall=\frac{\mathrm{TP}}{\left(\mathrm{TP}\;+\;\mathrm{FN}\right)}$.
F1 score	$\mathrm{Measure}\;\mathrm{indicates}\;\mathrm{the}\;\mathrm{harmonic}\;\mathrm{mean}\;\mathrm{of}\;\mathrm{precision}\;\mathrm{and}\;\mathrm{recall},\;F1\;\mathrm{score}=\frac{2*\mathrm{Precision}*\mathrm{Recall}}{\mathrm{Precision}\;+\;\mathrm{Recall}}$.
Specificity/TNR	$\mathrm{Measure}\;\mathrm{indicates}\;\mathrm{the}\;\mathrm{percentage}\;\mathrm{of}\;\mathrm{the}\;\mathrm{correct}\;\mathrm{negative}\;\mathrm{predictions},\;Specificity=\frac{\mathrm{TN}}{\left(\mathrm{TN}\;+\;\mathrm{FN}\right)}$.
AUC	The area under the curve (AUC) is a total measure of a binary classifier’s performance over all potential threshold settings.
MCC	$\;\mathrm{Matthews}\;\mathrm{correlation}\;\mathrm{coefficient}\;\mathrm{shows}\;\mathrm{the}\;\mathrm{true}\;\mathrm{positive}\;\mathrm{rate}\;(\mathrm{TPR})\;\mathrm{against}\;\mathrm{the}\;\mathrm{false}\;\mathrm{positive}\;\mathrm{rate}\;(\mathrm{FPR})\;\mathrm{for}\;\mathrm{various}\;\mathrm{threshold}\;\mathrm{values}.\;\;\mathrm{MCC}=\frac{\mathrm{TP}*\mathrm{TN}-\mathrm{FP}*\mathrm{FN}}{\left(\mathrm{TP}\;+\;\mathrm{FP}\right)\left(\mathrm{TP}\;+\;\mathrm{FN}\right)\left(\mathrm{TN}\;+\;\mathrm{FP}\right)\left(\mathrm{TN}\;+\;\mathrm{FN}\right)}$.
IoU	Intersection over union (IoU) is an object detection metric that finds the difference between ground truth annotations and predicted bounding boxes.
Error	Error is a measure that indicates the percentage of incorrect predictions, Error=1 − Accuracy.
Kappa	Kappa is an interesting metric used to measure classification performance.
ROC AUC/ROC	The receiver operating characteristic curve is a plot that shows the true positive rate (TPR) against the false positive rate (FPR) for various threshold values.
PR AUC/Average Precision	PR AUC is the average of precision scores calculated for each recall threshold.
NPV	$\mathrm{Negative}\;\mathrm{predictive}\;\mathrm{value}\;\mathrm{measures}\;\mathrm{how}\;\mathrm{many}\;\mathrm{predictions}\;\mathrm{out}\;\mathrm{of}\;\mathrm{all}\;\mathrm{negative}\;\mathrm{predictions}\;\mathrm{were}\;\mathrm{correct}.\;\mathrm{NPV}=\frac{\mathrm{TN}}{\mathrm{TN}\;+\;\mathrm{FN}}$.
FPR	$\mathrm{False}\;\mathrm{positive}\;\mathrm{rate},\;\mathrm{FPR}=\frac{\mathrm{FP}}{\left(\mathrm{FP}\;+\;\mathrm{TN}\right)}$.
FNR	$\mathrm{False}\;\mathrm{negative}\;\mathrm{rate},\;\mathrm{FNR}=\frac{\mathrm{FN}}{\mathrm{TP}\;+\;\mathrm{FN}}$.
NPR	False positive rate measures among truly negative cases to determine what percentage of them are actually false positive.
LRP	Localization recall precision is an error metric used to evaluate all visual detection tasks.

**Table 4:** Summary of deep learning segmentation methods used in the relevant publications in this review.

References	Data Set	Modalities	No. of Images	Partitioning	Classifiers	Performances (%)
[42]	Italian Society of Medical and Interventional Radiology	CT	1001 lung CT images	Training (72%) Validation (10%) Testing (18%)	SegNet U-NET	SegNet Sensitivity 0.956 Specificity 0.9542 U-NET Sensitivity 0.964 Specificity 0.948
(Paluru, N., Dayal, A., Jenssen, H.B., Sakinis, T., Cenkeramaddi, L.R., Prakash, J. and Yalavarthy, P.K, 2021) [43]	Italian Society of Medical and Interventional Radiology and Radiopedia	CT	929 lung CT images	Training (70%) Testing (30%)	Anam Net	Sensitivity 0.927 Specificity 0.998 Accuracy 0.985
(Yin, 2022) [44]	The Italian Society of Medical and Interactive Radiology	CT	1963 lung CT images	Training (1376 CT images) Validation (196 CT images) Testing (391 CT images	SD-Unet	Sensitivity 0.8988 Specificity 0.9932 Accuracy 0.9906
(Shan, et al., 2021) [45]	Shanghai Public Health Clinical Center and other centers outside of Shanghai	CT scan images	249 images	Training (75%) Testing (25%)	DL-based segmentation system (VB-Net)	Accuracy 0.916
[46]	Integrative Resource of Lung CT Images and Clinical Features (ICTCF) Med-Seg (medical segmentation) COVID-19 dataset	CT	7586 lung CT images	Training (698 CT images) Validation (6654 CT images) Testing (117 CT images)	SSInfNet	F1 score 0.63 Recall 0.71 Precision 0.68
[47]	Private dataset	CT	5000 CT images	Training (40%) Testing (60%)	COVLIAS 1.0 (SegNet, VGG-SegNet and ResNet-SegNet)	AUC: SegNet 0.96 VGG-SegNet. 0.97 ResNet-SegNet 0.98
[48]	Multiple sources of datasets	CT	4449 CT images	Training (4000 CT images) Testing (449 CT images)	ResUnet	Dice metric 72.81

**Table 5:** COVID-19 binary classification using a deep learning-based pre-trained model and deep transfer learning.

Authors	Data Sources	No. of Images	Name of Classes	Partitioning	Techniques	Performances (%)
[49]	[52,53]	1000 chest X-ray and CT images (normal = 805, COVID-19 = 195 (23 lung CT, 172 chest X-ray)	COVID-19, Normal	Training = 80% Test = 20%	VGG16, VGG19, Xception, ResNet50V2, MobileNetV2, NASNetMobile, ResNet101V2, and InceptionV3	Accuracy = 99% Sensitivity = 97.4% Specificity = 99.4%.
[42]	[54]	100 CT images	Infected, non-infected	Training = 70% Validation = 10% Test = 20% 5-fold cross validation	SegNet, U-NET	Accuracy = 95% Sensitivity = 95.6% Specificity = 95.42% Dice = 74.9% G-mean = 95.5% F2 = 86.1%
[50]	X-ray COVID-19 dataset [55]	50 X-ray images (COVID = 25, Normal = 25)	COVID, Normal	Training = 80% Test = 20% 5- and 10-fold cross validation.	ResNet50	5-folds cross validation: Accuracy = 97.28%. Precision = 96% Sensitivity = 96% F-measure = 96% 10-folds cross validation: Accuracy = 95.99% Precision = 95.83% Sensitivity =92% F-measure = 93.87%
[51]	Development dataset [56], Testing dataset: Zhejiang Province, China, lung segmentation development: El-Camino Hospital (CA), lung segmentation development: University Hospitals of Geneva (HUG).	1865 CT (normal = 1036, abnormal = 829)	Normal, COVID-19	Training = 1725 Validation = 320 Test = 270	ResNet-50-2D	AUC = 99.4% Sensitivity = 94% Specificity = 98%

**Table 6:** Summary of deep learning based COVID-19 binary classification using custom models.

Authors	Data Sources	No. of Images	Name of Classes	Partitioning	Techniques	Performances (%)
[57]	Local hospitals	640 CT (COVID-19 = 320, healthy controls (HCs) = 320	COVID-19, HC	10-fold cross validation	5LDCNN-SP-C	Sensitivity = 93.28% ± 1.50% Specificity = 94.00% ± 1.56% Accuracy = 93.64% ± 1.42%
[58]	data collection from Mendeley [52], The Cancer Imaging Archive (TCIA) [74], collection of X-rays and CT images that are COVID-19 positive [75]	753 X-ray images (COVID-19 = 253, normal = 500)	COVID-19, Normal	Train = 653: 5-fold cross validation Hold out = 100	CNN	Hold out test: Precision = 99% Recall = 99% F1 score = 99% AUC = 99% MCC = 99%
[59]	COVID-ct-dataset [76], Guangxi Medical University hospitals	2592 CT images (COVID-19 = 1357, non-infected = 1235)	COVID-19, non-infected	Training = 1867 Validation = 1400 Test = 510	Modified ResNet50	Specificity = 92% Sensitivity = 93% Accuracy = 93% IoU = 0.85 F1 score = 92% AUC = 93%
[60]	IOT		COVID, non-COVID	Training = 70% Validation = 30%	ID2S-COVID19-DL	Accuracy = 95.5% Sensitivity = 94.38% Specificity =97.06% Miss rate =1.89% PPV = 98.51% NPV = 97.62% FPR = 54.46% NPR = 0.02% LRP = 97.61% LRN = 98.51%
[61]	Open-source dataset [53], dataset from Kaggle [62]	574 CXR images (COVID = 287, viral and bacterial pneumonia = 287)	COVID, non-COVID	Training = 80% leave-Out = 20%	TDA-Net	Accuracy = 93% Precision = 88% Recall = 95% F1 score = 92% AUC = 100% TNR = 91%
[63]	Dataset collected from 3 centers: Xi’an Jiaotong University First Affiliated Hospital (center 1), Nanchang University First Hospital (center 2), Xi’an No.8 Hospital of Xi’an Medical College (center 3)	1065 CT images (COVID-19, typical pneumonia)	COVID-19, typical pneumonia	Training = 320 Internal Validation = 455 External validation = 290.	Modified Inception	Accuracy = 79.3% Specificity = 83% Sensitivity = 67%
[64]	COVID-CTset [77]	63,849 CT scan images (normal = 48,260, COVID-19 = 15,589)	COVID-19, normal	5-fold cross validation	ResNet50V2 + FPN	Accuracy = 98.49%
[65]	Open source repository provided by [53,78]	100 patients (50 COVID-19, 50 normal)	COVID-19, normal	k-fold cross validation (k = 5 and k = 10-fold)	ResNet101 + J48	k = 5-fold cross validation: Accuracy = 97.18% Recall = 98.64% Specificity = 95.86% Precision = 98.64% F1 score = 97.05% k = 10-fold cross validation: Accuracy = 100% Recall = 100% Specificity = 98.89% Precision = 100% F1 score = 100%
[66]	public COVID-19 CT dataset [76], Public pneumonia dataset [78],	public pneumonia dataset: 5856 X-ray images (normal and pneumonia) public COVID-19 CT dataset: 746 CT images (normal and pneumonia)	Pneumonia, normal	Public pneumonia dataset: Training = 5216 Validation = 16 Testing = 624 public pneumonia dataset: Training = 425 Validation = 118 Testing = 203	CGNet	Public pneumonia dataset: Accuracy = 98.72% Sensitivity = 100% Specificity = 97.95% Public COVID-19 CT dataset: Accuracy = 99% Sensitivity = 100% Specificity = 98%
[67]	Sites the Northwestern Memorial Health Care System	15,035 CXR images (COVID-19 positive = 4750, COVID-19 Negative = 10,285)	COVID-positive, COVID-negative	Training = 10,470 validation = 2686 Testing = 1879	DeepCOVID-XR	For the entire test set: Accuracy = 83% AUC = 90% For 300 random test images: Accuracy = 82%
[68]	Dataset includes CT images [79], dataset includes X-ray images [80], COVID-19 radiography dataset [81]	6130 images (COVID-19 = 3065, non-COVID-19 = 3065)	COVID-19, viral pneumonia	Training = 70% Test = 30%	CNN + ConvLSTM	Accuracy = 100%
[69]	Multiple sources [53,54,62,80,82]	4600 X-ray images (COVID-19 = 2300, Normal = 2300)	COVID-19, normal	Training = 70% Validation = 20% Test = 10%	EMCNet	Accuracy = 98.91% Precision = 100% Recall = 97.82% F1 score = 98.89%
[70]	Two open-source image databases [53,78]	1365 chest X-ray images (COVID-19 = 250, normal = 315, Viral Pneumonia = 350, bacterial pneumonia = 300, Other = 150)	COVID-19, other	Training = 70% Validation = 20% Test = 10% 5-fold cross validation	ResNet50 + ResNet-101	Accuracy = 97.77% Recall = 97.14% Precision = 97.14% With cCross validation: Accuracy = 98.93% Sensitivity = 98.93% Specificity = 98.66% Precision = 96.39% F1 score = 98.15%
[71]	Joseph Paul Cohen dataset [53], Publicly available dataset [78],	5216 chest X-ray and CT images (normal = 1341, pneumonia = 3875)	COVID-19, normal	Training = 80% Test = 20%	IRRCNN	X-ray images: Accuracy = 84.67% CT images: Accuracy = 98.78%
[72]	Archiving and communication system (PACS) of the radiology department (Union Hospital, Tongji Medical College, Huazhong University of Science and Tech)	540 CT images (COVID-positive = 313, COVID-negative = 229)	COVID-positive, COVID-negative	Training = 499 Test =131	DeCoVNet	ROC AUC = 95.9% PR AUC = 97.6% Sensitivity = 90.7% Specificity = 91.1%
[73]	COVID-19 CT dataset [76]	738 CT images (COVID = 349, non-COVID = 463)	COVID, non COVID	Training = 80% Validation = 10% Test = 10%	CTnet-10	Accuracy = 82.1%

**Table 7:** Summary of deep learning based COVID-19 multi-classification using pre-trained model with deep transfer learning.

Authors	Data Sources	No. of Images	Name of Classes	Partitioning	Techniques	Performances (%)
[83]	Two Kaggle datasets [4,92], COVID-19 image data collection [53]	1491 chest X–rays and CT scans (normal = 1335, mild/moderate = 106, severe = 50)	Normal, mild/moderate, Severe	Training = 70% Validation = 15% Test = 15%	AlexNet GoogleNet Resnet50	Average accuracy (non-augmented) AlexNet 81.48% GoogleNet 78.71% Resnet50 82.10% Average accuracy (augmented) AlexNet 83.70% GoogleNet 81.60% Resnet50 87.80%
[84]	BIMCV COVID-19 dataset [93], PadChest dataset [94]	11,197 CXR (Control = 7217, pneumonia = 5451, COVID-19 = 1056)	Control, pneumonia, COVID-19	Training = 70% Validation = 15% Test = 15%	DenseNet161	Average balanced accuracy = 91.2%, Average precision = 92.4% F1 score = 91.9%
[85]	COVIDx dataset [95]	15,177 Chest X-ray images (COVID-19 = 238, pneumonia = 6045, Normal = 8851)	COVID-19, non-COVID- COVID-19, pneumonia, normal	Training = 80% Validation = 10% Test = 10% 10-fold cross validation	DenseNet-121	Two-class: Accuracy = 96% Precision = 96% Recall = 96% F-score = 96% Three-class: Accuracy = 93% Precision = 92% Recall = 92% F-score = 92%
[86]	Public dataset of X-ray images collected by [53]	306 X-ray images (normal = 79, COVID-19 = 69, viral pneumonia = 79, bacterial pneumonia = 79)	Normal, COVID-19, viral pneumonia, bacterial pneumonia	Training = 85% Test = 15%	Cascaded deep learning classifiers (VGG16, ResNet50V2, DenseNet169)	Accuracy = 99.9%
[87]	[53,78]	673 X-ray and CT images (COVID-19 = 202, normal = 300, pneumonia = 300)	COVID-19, pneumonia, normal	Training = 80% Test = 20%	VGG-16, ResNet50, EfficientNetB0	Accuracy = 96.8%
[88]	Multiple sources [52,53,81,96]	11568 X-ray images (COVID-19 = 371, non-COVID-19 viral pneumonia = 4237, bacterial pneumonia = 4078, normal = 2882)	COVID-19, viral pneumonia, bacterial pneumonia, normal	Training = 70% Test = 30%	AlexNet	Accuracy = 99.62% Sensitivity = 90.63% Specificity = 99.89%.
[89]	Kaggle repository [97]	6432 (COVID-19 = 576, pneumonia = 4273, normal = 1583)	COVID-19, pneumonia, normal	Training = 5467 Validation = 965	CNN models: Inception V3 Xception ResNeXt	Accuracy = 97.97%
[90]	chest X-ray dataset [53], RSNA pneumonia dataset [98]	18,567 (COVID-19 = 140, viral pneumonia = 9576, normal = 8851)	COVID-19, viral pneumonia, normal	Training = 16714 Test = 1862	ResNet101 ResNet152	Accuracy = 96.1%
[91]	Publicly available image datasets (chest X-ray and CT dataset) [52,53]	6087 chest X-ray and CT images (bacterial pneumonia = 2780, coronavirus = 1493, COVID19 = 231, normal = 1583)	Normal, bacteria, coronavirus	Training = 80% Validation = 20%	VGG16, VGG19, DenseNet201, Inception_ResNet_V2, Inception_V3, Resnet50, MobileNet_V2	Accuracy = 92.18%

**Table 8:** Summary of deep learning based COVID-19 multi-classification using custom models.

Authors	Data Sources	No. of Images	Name of Classes	Partitioning	Techniques	Performances (%)
[99]	Journals: Science direct, Nature, Springer Link, and China CNKI, Thoritative media reports: New York Times, Daily Mail (United Kingdom), The Times (United Kingdom), CNN, etc.	2933 lung CT images	COVID, lung tumor, normal lung	Training = 6000 Test =1500 5-fold cross validation.	EDL-COVID	Accuracy = 99.054%. Sensitivity = 99.05% Specificity = 99.6% F measure = 98.59% MCC = 97.89%
[100]	Multiple sources [4,53,81,98,118]	13,975 CXR images (normal = 7966, pneumonia = 5451, and COVID-19 pneumonia = 258)	Healthy, pneumonia, COVID-19	Training = 13,675 Test = 300	Modified COVID-net	Accuracy = 94.3% Sensitivity = 94.3% ± 4.5% Specificity = 97.2% ± 1.9% PPV = 94.5% ± 3.3% F score = 94.3% ± 2.0%
[13]	Two open-source datasets [52,53]	15,085 X-ray (normal = 8851, COVID-19 = 180, pneumonia = 6054)	Normal, COVID-19, pneumonia	cross entropy 3-fold cross validation	Modified ResNet18	Accuracy = 96.73% Recall = 94% Specificity = 100%
[101]	COVID-19 CXR dataset [53], Xiangya Hospital RSNA pneumonia detection challenge [98]	3545 chest X-ray images (COVID-19 = 204, healthy = 1314, CAP = 2004)	COVID-19, Healthy, CAP	Training = 80% Validation = 20% Test = 61 images	ResNet50 + FPN	Accuracy = 93.65% Sensitivity = 90.92% Specificity = 92.62%
[102]	Two Kaggle datasets [53,92]	1389 X-ray images (COVID-19 = 289, viral pneumonia = 550, normal = 550)	COVID-19, viral pneumonia, normal	5-fold cross validation	CNN	Accuracy = 90.64% F1 score = 89.8%
[103]	Open-access database [4]	2905 CXR images (COVID-19 = 219, viral pneumonia = 1345, normal = 1341)	COVID-19, viral pneumonia, normal		mAlexNet	Accuracy = 98.70% Error = 0.0130 Recall = 98.76% Specificity = 99.33% Precision = 98.77% False positive rate = 0.0067 F1 score = 98.76% AUC = 99.00% MCC = 98.09% Kappa = 97.07%
[104]	COVID-19 Radiography Database [4], Chest X-ray dataset [119]	3047 chest X-ray images (COVID-19 = 361, pneumonia = 1341, normal = 1345)	COVID, non-COVID COVID-19, pneumonia, normal	Training = 80% Test = 20%	InstaCovNet-19	Two class: Accuracy = 99.53% Precision = 100% Recall = 99% Three class: Accuracy = 99.08% Recall = 99% F1 score = 99% Precision = 99%
[105]	Multiple sources [53,54,78,82,98,118,120]	15,265 chest X-ray images (COVID-19 = 558, normal = 10,434, bacterial pneumonia = 2780, Viral pneumonia = 1493)	COVID-19, normal, viral pneumonia, bacterial pneumonia	5-fold cross validation	CSDB CNN	Precision = 96.34 Recall = 97.54% F1 score = 96.90% Accuracy = 97.94% Specificity = 99.25% AUC = 98.39%
[106]	COVID-19 dataset [53], chest-X-ray images [78]	CXR (COVID-19 = 145, Bacterial Pneumonia = 145, normal = 145)	COVID, non-COVID COVID, non-COVID, bacterial pneumonia	Training = 80% Test = 20%	deep learning conditional generative adversarial networks	Two class: Accuracy = 98.7% Sensitivity = 100% Specificity = 98.3% Three class: Accuracy = 98.3% Sensitivity = 99.3% Specificity = 98.1%
[107]	Multiple sources [4,52,53]	1092 X-ray images (COVID-19 = 364, normal 364, pneumonia = 364)	COVID-19, normal COVID-19, normal, pneumonia	Training = 70% Test = 30% 5-fold cross validation	MH-COVIDNet	Accuracy = 99.38%
[108]	Multiple sources [4,53,79,92,118,120,121,122]	7390 X-ray and CT images (COVID-19 = 2843, normal = 3108, viral pneumonia + bacterial pneumonia = 1439)	COVID, normal COVID, normal, pneumonia COVID, normal, viral pneumonia, bacterial pneumonia	5-fold cross validation	CoroDet	Two class: Accuracy = 99.1% Sensitivity = 95.36% Specificity = 97.36% Precision = 97.64% Recall = 95.3% F1 score = 96.88% Three class: Accuracy = 94.2% Sensitivity = 92.76% Specificity = 94.56% Precision = 94.04% Recall = 92.5% F1 score = 91.32% Four class: Accuracy = 91.2% Sensitivity = 91.76% Specificity = 93.48% Precision = 92.04% Recall = 91.9% F1 score = 90.04
[24]	LUNGx Challenge for computerized lung nodule classification [123]	16,750 CT images (COVID-19 = 5550, CAP = 5750, control = 5450)	COVID-19, Non-COVID COVID-19, CAP, control	Training = 15,000 Validation = 750 Test = 1000	COVIDCTNet	Sensitivity = 93% Specificity = 100% Two class: Accuracy = 95% Three class: Accuracy = 85%
[109]	COVID-19 dataset [53]	1184 chest X-ray images (COVID-19 = 336, MERS = 185 SARS = 141, ARDS = 130, Normal = 392)	COVID-19, MERS, SARS, ARDS, normal	Training = 757 Test = 427	CNN	Accuracy = 98% Precision = 99% Recall = 98% F1 score = 98%
[110]	Multiple sources [53,81,92,118,122,124,125]	6317 chest X-ray images (COVID-19 = 1440, normal = 2470 viral and bacterial pneumonia = 2407)	COVID-19, normal, pneumonia	Training = 70% Test = 30%	Convid-Net	Accuracy = 97.99%
[111]	COVID-19 Image Data Collection [53], RSNA Pneumonia Detection Challenge dataset [98], COVID-19 Chest X-ray Dataset Initiative [120]	13,862 chest X-ray samples (COVID-19 = 245, pneumonia = 5551, normal = 8066)	COVID-19, pneumonia, normal	Training = 20,907 Test = 231	Corona-Nidaan	For three-class classification: Accuracy = 95% For COVID-19 cases: Precision = 94% Recall = 94%
[112]	[78,126,127]	1061 CX images (COVID-19 = 361, normal = 200, pneumonia = 500)	COVID-19, pneumonia, normal	Training = 80% Testing = 20%	DeepCoroNet	Accuracy = 100% Sensitivity = 100% Specificity = 100% F score = 100%
[113]	Multiple sources [53,74,98,128]	10,377 X-ray and CT images (normal, pneumonia, COVID-19, influenza)	COVID-19, pneumonia, normal	Training = 9830 Test = 547	CNNRF	F1 score = 98.90% Specificity = 100%
[114]	Multiple sources [52,53,129,130]	6792 CXR images (normal = 1840, COVID-19 = 433, TB = 394, BP = 2780, VP = 1345)	COVID-19, normal, tuberculosis (TB), bacterial pneumonia (BP), viral pneumonia (VP)	Training = 80% Validation = 10% Test = 10%	MANet	Accuracy = 96.32%
[115]	COVID-19 dataset [4], Joseph Paul Cohen dataset [53]	458 X-ray images (COVID-19 = 295, pneumonia = 98, normal = 65)	COVID-19, pneumonia, normal	Training = 70% Test = 30% 5-fold cross validation	MobileNetV2 + SqueezeNet	Accuracy = 99.27%
[116]	X-VIRAL dataset collected from 390 township hospitals through a telemedicine platform of JF Healthcare, X-COVID dataset collected from 6 institutions, COVID-19 dataset [53]	Chest X-ray images (positive viral pneumonia = 5977, non-viral pneumonia or healthy = 37,393, COVID-19 = 106, normal controls = 107)	COVID, non-COVID COVID, SARS, MERS	5-fold cross validation	CAAD	X-COVID dataset: Two class AUC = 83.61% Sensitivity = 71.70% Open-COVID dataset: Three class Accuracy = 94.93% for COVID-19 detection Accuracy = 100% for SARS and MERS detection
[117]	COVID-19 Radiography Database [4]	2905 chest X-ray images (COVID-19 = 219, viral pneumonia = 1341, normal = 1345)	COVID, viral pneumonia, normal	5-fold cross validation Training = 70% Validation = 10% Test = 20%	CVDNet	Precision = 96.72% Accuracy = 96.69% Recall = 96.84% F1 score = 96.68% Accuracy = 97.20% for COVID-19 class

## Data Availability

Not applicable.
